# The role of circulating tumor cell-associated genes in the progression of estrogen receptor-positive breast cancer

**DOI:** 10.1038/s41523-025-00874-0

**Published:** 2025-12-15

**Authors:** Martin Rotbauer, Melanie Dawe, Kevin C. J. Nixon, Julissa Tsao, Tanja Durbic, Monika Sharma, Sheng-Ben Liang, Jie Zhang, Nakita E. K. Gopal, Miralem Mrkonjic, Najd Alshamlan, Adnan Karavelic, Ciara Murray, David W. Cescon, Philippe L. Bedard, Susan J. Done

**Affiliations:** 1 Princess Margaret Cancer Centre, University Health Network, Toronto, Canada; 2 Department of Laboratory Medicine and Pathobiology, University of Toronto, Toronto, Canada; 3 Princess Margaret Genomics Centre (PMGC), University Health Network, Toronto, Canada; 4 Princess Margaret Cancer Biobank (PMCB), University Health Network, Toronto, Canada; 5 Laboratory Medicine Program, University Health Network, Toronto, Canada; 6 Pathology and Laboratory Medicine, Mount Sinai Hospital, Toronto, Canada; 7 Division of Medical Oncology & Hematology, Department of Medicine, University Health Network, Toronto, Canada

**Keywords:** Genetics, Molecular biology, Cancer, Cancer genetics, Cancer genomics, Tumour heterogeneity, Biomarkers, Prognostic markers, Oncology, Cancer

## Abstract

Estrogen receptor-positive, human epidermal growth factor receptor negative (ER + /HER2−) breast cancer, poses challenges in adjuvant treatment decisions due to its propensity for late recurrence. We propose a model that leverages our previously identified circulating tumor cell (CTC) genomic signature, linked to metastasis. Furthermore, we investigated the impact of CTC signature intratumour heterogeneity (ITH) on recurrence risk. Using Oncotype DX recurrence score as a surrogate for survival, we trained expression and copy number-based models using 194 early stage ER + /HER2− breast cancer patients and validated them in the METABRIC dataset. Multispectral fluorescence in situ hybridization (Multiplex-FISH) was used to evaluate the ITH of 6 CTC genomic regions in primary tumors. The expression-based model strongly correlated with Oncotype DX, while the copy number-based model achieved a moderate correlation. Both models were able to predict long-term recurrence free survival in METABRIC. Higher CTC signature ITH was associated with increased Oncotype DX risk and higher overall grade. These findings highlight the value of our CTC signature in disease progression and the role of ITH on recurrence risk.

## Introduction

Estrogen receptor positive (ER+), human epidermal growth factor receptor negative (HER2−) breast cancer is the most diagnosed subtype of breast cancer representing approximately 75% of all cases^1,2^. Although it has the highest survival rate among all the molecular subtypes, ER+ breast cancer has a tendency for late recurrence^2,3^. It has been shown to recur even 15 years after an initial 5-year disease-free period in patients treated with adjuvant endocrine therapy^4^. Patients with an increased risk for early recurrence typically receive chemotherapy in addition to endocrine therapy but, in some cases, accurately estimating this risk can be challenging from clinicopathological features alone, leading to overtreatment^5–7^. The Oncotype DX Breast Recurrence Score Test (Exact Sciences, formerly Genomic Health) is a multigene prognostic assay comprising a multiplex RT-PCR analysis of 16 cancer-related genes and 5 reference genes, developed to assist treatment decisions in ER + /HER2− breast cancer patients^8^. The assay assigns a recurrence score between 0-100 with predefined risk thresholds that inform adjuvant treatment. Since its introduction in 2004, Oncotype DX has established itself as a gold standard test in its class, through extensive prospective clinical trials including the Trial Assigning Individualized Options for Treatment (TAILORx) and RxPONDER^9,10^. TAILORx which enrolled close to 10,000 women found that Oncotype DX can accurately classify patients into risk groups with clear implications for adjuvant treatment. RxPONDER expanded the significance of the assay to predict recurrence in lymph node positive ER+ cases, a cohort that was not included in previous studies. The Oncotype DX although robust for early recurrence prediction is not as accurate in predicting late recurrence, a hallmark of ER + /HER2− cancer which has recurrence rates up to 20 years after initial diagnosis^11,12^. Other multigene prognostic tests approved for use in ER + /HER2− breast cancer such as MammaPrint, Prosigna PAM50, Endopredict and the Breast Cancer Index exist but are generally less widely used in routine clinical practice in North America, and are approved to estimate recurrence up to 5–10 years, similar to Oncotype^13,14^.

Distant breast cancer recurrence, including late recurrence in ER + /HER2− disease, can be attributed to circulating tumor cells (CTCs). CTCs seed metastasis, and their enumeration and heterogeneity have direct implications for survival and treatment in breast cancer^15–19^. The utilization of CTCs for prognosis in liquid biopsy has long held promise in oncology, driven by numerous potential advantages, including minimal invasiveness and the capability for real-time disease monitoring^20–22^. However, significant challenges, such as identifying reliable CTC markers and improving the sensitivity of CTC detection, are barriers to the implementation of CTC liquid biopsy as a clinical tool^20–22^. An alternative approach, which we have explored previously, is to evaluate the genomic and phenotypic features that characterize CTCs in primary tumors. We have developed a signature of genes that are frequently altered in CTCs from breast cancer patients and assessed the intra-tumoral heterogeneity (ITH) and frequency of this signature in pre and post-treatment primary and metastatic breast tumors. We found in treatment naïve tumors, those with a higher ITH for the CTC signature had a worse prognosis^23,24^. Additionally, our CTC signature was significantly enriched in post treatment metastatic tumors compared to their matched pre-treatment primaries^24^. This suggests that the CTC signature has a role in treatment resistance, metastasis, and recurrence.

Based upon these findings, we hypothesized that the CTC signature may have potential for predicting recurrence risk in ER + /HER2− breast cancer. Our aim was to investigate if expression, copy number, and ITH of our signature correlates with recurrence, using the Oncotype DX score as a surrogate for recurrence risk.

## Results

### Characteristics of Patients

**Fig. 1:**
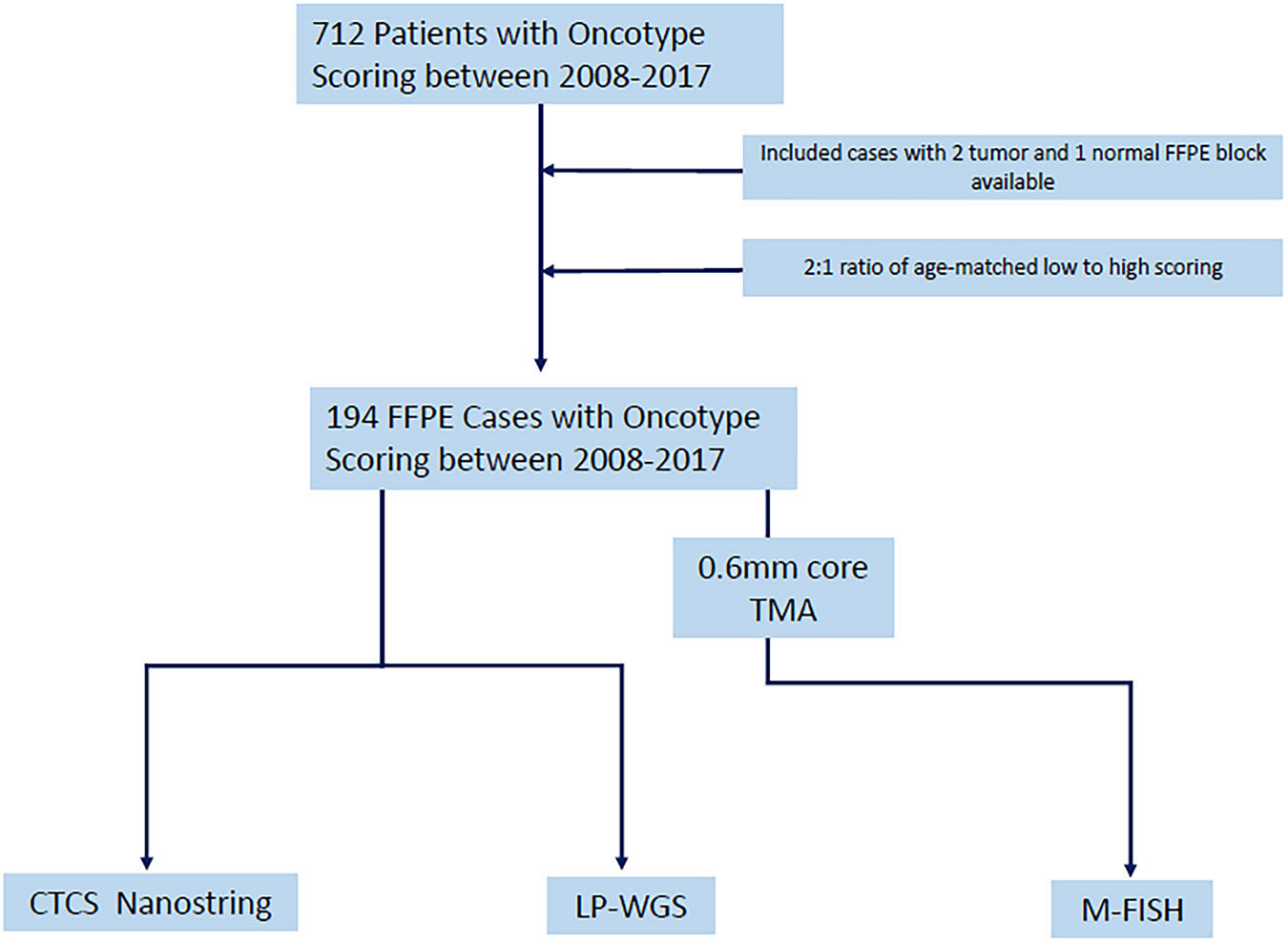
Study design and sample selection.

A total of 712 patients had Oncotype DX testing between the years of 2008 and 2017 ranging in scores from 0 to 70 (mean: 21.8, median: 20). For simplicity and due to the average age of the cohort, we chose to dichotomize scores into two groups using the TAILORx guidelines for postmenopausal scoring^10^. Cases were selected by a 2:1 ratio of age-matched low-scoring (<26) to high-scoring (≥26) patients. This distribution was selected to reflect that clinically more patients present with a low Oncotype DX score, while ensuring that the cohort also has a good representation of high-risk patients. Additionally, for eligibility, cases needed to have two tumor and one adjacent normal block available with enough material to undergo DNA and RNA extraction as determined by an experienced breast pathologist (Fig. 1). In total 194 cases were selected and eligible for further analysis. The overall grade ranged from I to III (score 5/9 to 9/9, mean: 7.2/9, median: 7/9). At the time of the study, 11 of 194 patients had experienced cancer recurrence in a mean and median time of 4.9 and 5.1 years, respectively, and 190 were alive. Clinical features of the 194 cohort are included in Table 1.

**Table 1:** Clinical and Pathological Features of 194 patients

Characteristic	Low Oncotype Score (<26, n = 130)	High Oncotype Score (≥26, n = 64)
Median Age (range)	56(35–78)	57.5(32–80)
Tumor Size (cm)		
Median (range)	1.7 (0.7-3.7)	1.7 (0.8-3.7)
Mean	1.88	1.82
Overall Histological Grade (%)		
I	1.5	1.6
II	65.4	42.2
III	33.1	56.2
Median Estrogen Receptor Percent (range)	99 (70-100)	98 (1-100)
Median Progesterone Receptor Percent (range)	80 (0-100)	15(0-95)
Lymph Node Involvement (%)		
No	89.2	92.2
Yes	10.8	7.8
Surgery Type (%)		
Mastectomy	18.5	17.2
Lumpectomy	81.5	82.8
Recurrence (%)		
No	95.4	92.2
Yes	4.6	7.8
Survival Event (%)		
Alive	97.7	98.4
Deceased	2.3	1.6

### CTC signature expression-based recurrence test

**Fig. 2:**
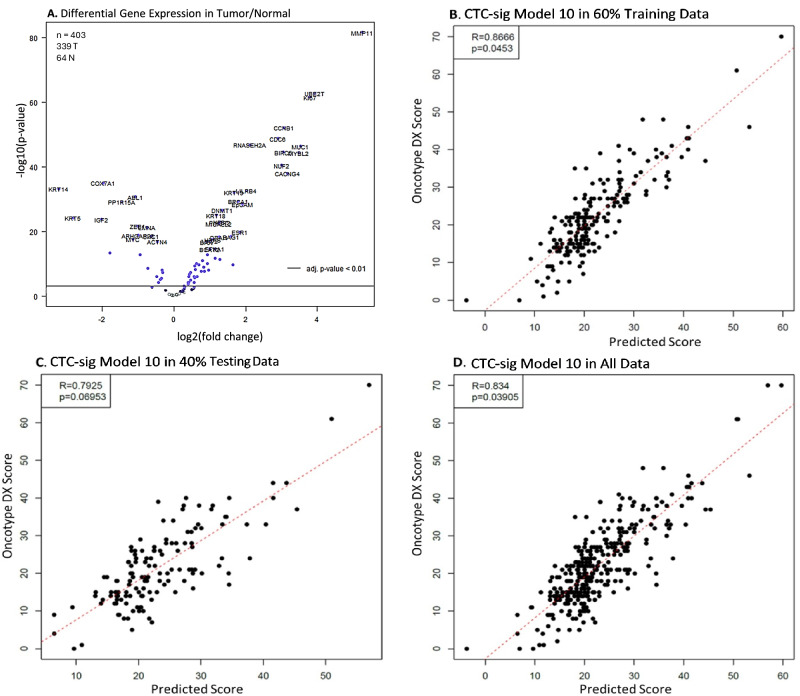
CTC signature gene expression-based recurrence model. **A** Volcano plot of CTC gene panel differential expression in 339 tumor samples compared to 64 matched normals for baseline. Significance threshold of *p* < 0.01 is indicated. **B–D** LASSO expression model trained on 203 tumor samples (60% training data) and validated on 136 tumor samples (40% testing data) and all samples (*n* = 339) against Oncotype DX scores. Pearson correlations of: R = 0.87 (*p* = 0.045), R = 0.79 (*p* = 0.070), and R = 0.83 (*p* = 0.039) were achieved in the training, testing, and all data, respectively.

**Fig. 3:**
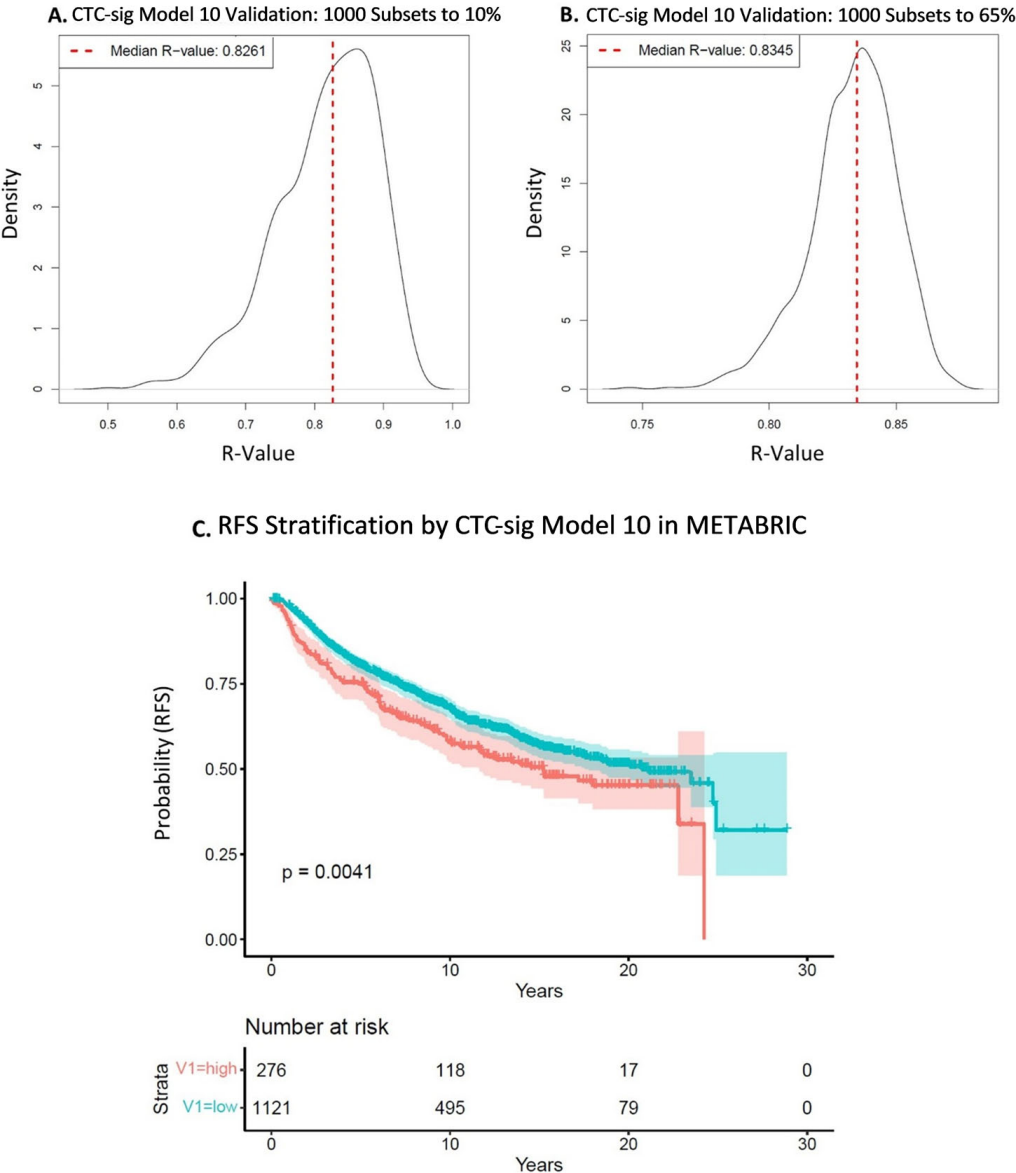
LASSO expression CTC-sig model 10 validation. **A**, **B** Validation in tumor sample subsets. One thousand random subsets, ranging from 10% to 100% of all tumor samples, were generated to assess model performance. Distributions of R values for 1000 subsets at 10% and 65% are displayed, with median R indicated by a dotted line. **C** Kaplan-Meier curve of LASSO expression CTC-sig model 10 in the METABRIC dataset (all patients). The model, applied without coefficients, stratified patients into low (turquoise) and high (orange) risk groups. Shaded areas represent a 95% confidence interval, and the log-rank *p*-value is 0.0041.

The expression of our CTC signature gene panel was evaluated with a custom NanoString nCounter CodeSet in 339 tumor and 64 matched normal samples from 194 ER + /HER2− breast cancer patients with Oncotype DX scores. Most genes were significantly differentially expressed in tumor versus normal samples, and more genes were overexpressed than underexpressed in tumors (*p* < 0.01) (Fig. 2A). Multiple iterations of LASSO regression were used to train a model against Oncotype DX scores in 60% of the tumor samples. The best model based on performance in training and testing data (model iteration #10) was selected. Model 10 (CTC-sig model 10) achieved a strong Pearson correlation of R = 0.87 (*p* = 0.045) in training data (Fig. 2B). In the unseen testing data, the model achieved a correlation of R = 0.79 (*p* = 0.070), and in all tumor samples, a correlation of R = 0.83 (*p* = 0.039) (Fig. 2C, D). The performance of the other models in 60% training and 40% testing data can be seen in Supplementary Figs. S1, S2. The selected CTC-sig model 10, consisting of 46 genes, can be found in Supplementary Table S1. To evaluate the robustness of CTC-sig model 10, we tested its performance in 1000 random subsets from 10 to 100% of all tumor samples. The model maintained a consistently strong positive correlation of R > 0.82 versus Oncotype DX score across all subset sizes. For example, the median R was 0.83 in 1000 random subsets to 10% as well as in 1000 random subsets to 65% (Fig. 3A, B). The corresponding median *p*-values were *p* = 0.13 and *p* = 0.048, respectively (Supplementary Fig. S3). External validation of CTC-sig model 10 was performed with the expression data of 1397 breast cancer patients in the METABRIC dataset, using the signs (+/−) without coefficients for each gene. There was a significant difference observed over 20 years in recurrence free survival (RFS) when patients were stratified by CTC-sig model 10 into low and high-risk groups (*p* = 0.0041) (Fig. 3C). At 10 years after surgery, the RFS rates were 68% (CI) and 58% (CI) in the low-risk and high-risk groups, respectively. Significant differences between risk groups were maintained when evaluating only ER + /HER2− METABRIC patients, and when separately evaluating the CTC signature genes versus non-CTC signature genes in model 10 (Supplementary Fig. S4). CTC signature genes steadily improved in their predictive ability over 20 years, while non-CTC signature genes had the best performance between 6 and 10 years after surgery. Collectively, these results suggest that a CTC signature-based gene expression has potential to predict recurrence risk over time.

### CTC signature copy number-based recurrence test

**Fig. 4:**
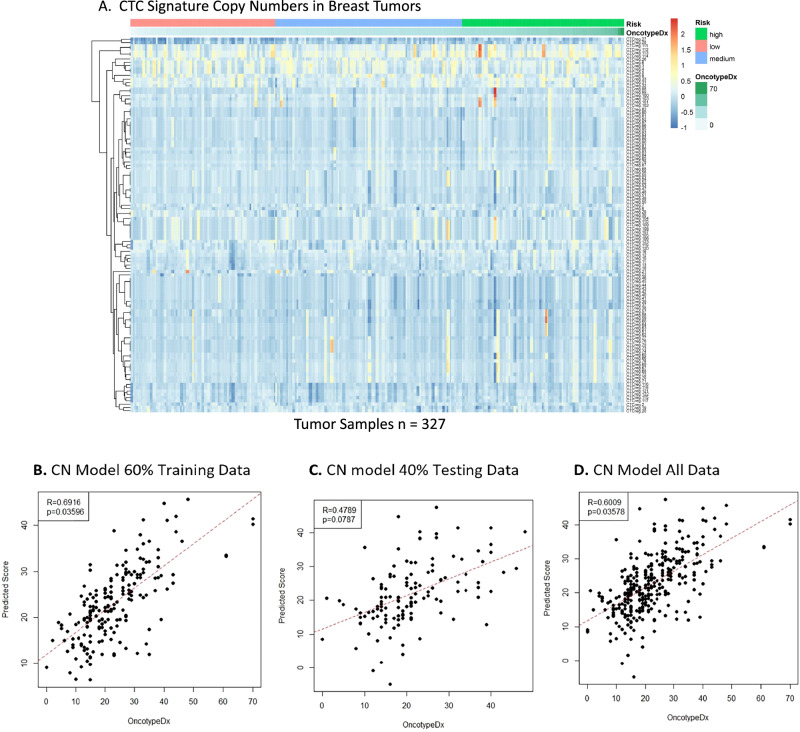
CTC Signature copy number model generation. **A** Heatmap of log ratio of copy number for 1 Mb CTC signature segments in tumor samples (*n* = 327) versus panel of normals (*n* = 55). Risk groups (low = pink, intermediate = blue, high = green) and Oncotype DX scores (white = lower, green = higher) are annotated in ascending order. **B–D** Stepwise feature selection copy number model. Tumor samples (*n* = 327) were divided into 60% training data (*n* = 196) and 40% testing data (*n* = 131) subsets. A stepwise feature selection model trained on CTC signature copy number regions against Oncotype DX scores in the training data was applied to the testing data and all data. Pearson R values of 0.69 (*p* = 0.036), 0.48 (*p* = 0.079), and 0.60 (*p* = 0.036) were achieved in the training data, testing data, and all data, respectively.

**Fig. 5:**
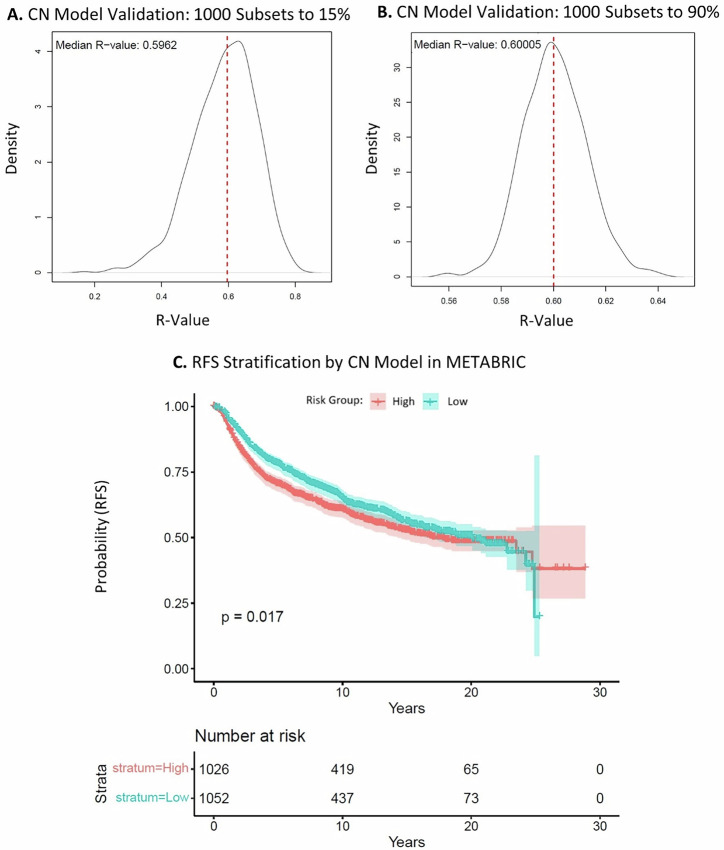
Copy number model validation. **A**, **B** Stepwise feature selection copy number model validation in tumor sample subsets. One thousand random subsets, ranging from 15% to 100% of all tumor samples, were generated to assess model performance. Distributions of R values for subsets at 15% and 90% are displayed, with median R indicated by a dotted line. **C** Kaplan-Meier curve of the stepwise feature selection copy number model in the METABRIC dataset. The model, applied to METABRIC copy number data using annotated genes from CTC regions, stratified patients into low (turquoise) and high (orange) risk groups. Shaded areas represent a 95% confidence interval, and the log-rank *p*-value = 0.017.

Currently available recurrence tests are expression-based. The CTC signature was discovered as a genomic signature of copy number gain. Therefore, we sought to also develop a copy number-based breast cancer recurrence test from our CTC signature. LP-WGS data for all samples was processed with ichorCNA to derive copy number calls for 1 Mb genomic regions. There was a strong positive correlation of copy number calls for 40 ng versus 100 ng sequences of the same samples: R = 0.87–0.99 (*p* < 0.001) (Supplementary Fig. S5). CTC signature genomic regions were mostly copy number neutral in tumor samples (*n* = 327) versus a panel of normal samples (*n* = 55), and no correlation of copy number to Oncotype DX score could be observed (Fig. 4A). Stepwise feature selection was used to develop a copy number model from CTC signature regions against Oncotype DX scores in 60% of tumor samples. Here, the stepwise copy number model achieved a Pearson correlation of R = 0.69 (*p* = 0.036) (Fig. 4B). In the remaining 40% of unseen tumor samples, the stepwise model achieved a correlation of R = 0.48 (*p* = 0.079), and in all tumor samples, a correlation of R = 0.60 (*p* = 0.036) (Fig. 4C, D). The stepwise copy number model performed consistently across all random subset sizes from 15 to 100% of all tumor samples, with a correlation of R ≈ 0.60. For example, the model achieved a median R value of 0.60 in both 1000 random subsets to 15% and 90% (Fig. 5A, B). The corresponding median *p*-values were *p* = 0.14 and *p* = 0.058, respectively (Supplementary Fig. S6). In METABRIC copy number data of 2078 breast cancer patients, there was a significant difference in RFS over 20 years when stratifying patients into low and high-risk groups using annotated genes from the model (*p* = 0.017) (Fig. 5C). At 10 years after surgery, the RFS rates were 66% and 61% in the low risk and high-risk groups, respectively. The full copy number models and conversions to genomic regions can be found in Supplementary Tables S2, S3. LASSO modeling was also performed; however, it did not yield results as significant as the stepwise feature selection approach (Supplementary Figs. S7–S9). Overall, our copy number-based recurrence model only moderately correlated to Oncotype DX recurrence score and did not perform as strongly as the expression-based CTC-sig model 10 at predicting RFS.

### CTC signature ITH as a recurrence predictor

**Fig. 6:**
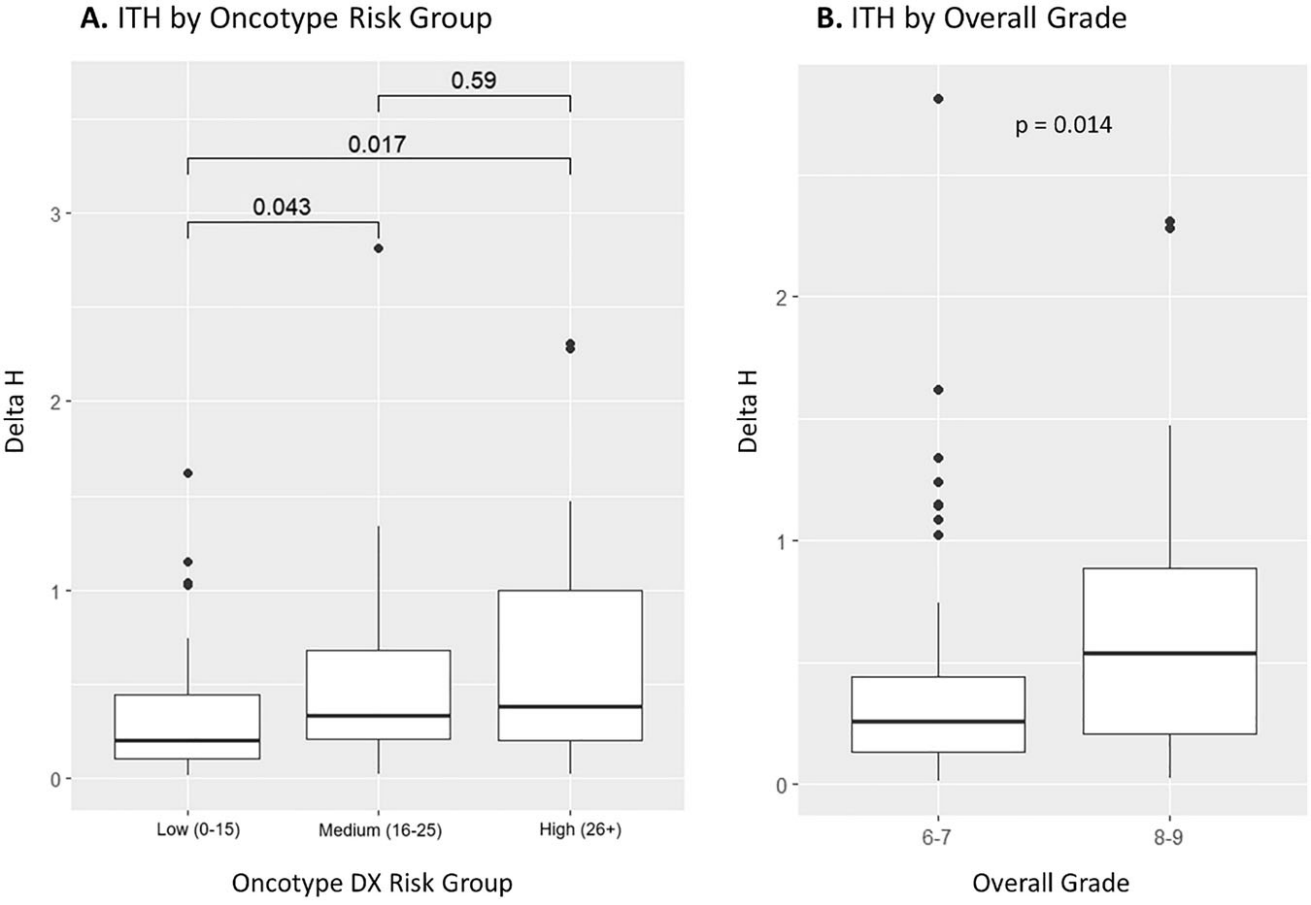
ITH score (∆H) by Oncotype DX risk group and overall grade. **A** Box plot of ∆H distribution across Oncotype DX risk groups. ∆H was compared among low(0-15), intermediate^16–25^, and high (26–100) risk groups, consisting of 33, 38, and 37 patients, respectively. Wilcoxon rank-sum tests revealed significant differences in ∆H scores between low and intermediate risk groups (*p* = 0.043), low and high-risk groups (*p* = 0.017), but not between intermediate and high-risk groups (*p* = 0.59). **B** Box plot of ∆H distributions based on overall grade. ∆H was compared between patients with an overall grade of II (score 6/7, *n* = 59) and III (score, 8/9, *n* = 49), showing a significant difference in ∆H scores (Wilcoxon rank-sum test *p* = 0.014).

We have previously demonstrated that ITH of the CTC signature is positively correlated to distant metastasis in breast cancer^24^. Here, we sought to investigate whether ITH could be directly correlated to recurrence risk assessment. An intratumour heterogeneity score ∆H was calculated for 108 patients from our Oncotype cohort who had 2 separate tumor cores that could be evaluated and passed cut-offs for inclusion based on a false positivity rate of 10.6% from matched normal samples. The mean and median ∆H score was 0.50 and 0.30, respectively, and the distribution was skewed to the right (Supplementary Fig. S10). When patients were stratified into low and high Oncotype DX risk groups, there was a significantly higher ∆H in the intermediate risk group (*n* = 38, median ∆H = 0.33) and high-risk group (*n* = 37, median ∆H = 0.38) compared to patients in the low risk group (*n* = 33, median ∆H = 0.20) (*p* < 0.05) (Fig. 6A). No significant difference was found between intermediate and high-risk groups. When patients were stratified by overall grade II (score 6/7, *n* = 59) and III (score 8/9, *n* = 49), respectively, there was a significantly higher ∆H in the group with a higher overall grade (median ∆H = 0.54 versus 0.26) (*p* = 0.014) (Fig. 6B).

## Discussion

The most common molecular subtype of breast cancer, ER + /HER2−, poses challenges in determining the need for adjuvant chemotherapy as it can recur up to 20 years or more after initial diagnosis^1–4,6,7^. While many patients with ER+ breast cancers are cured, it is important to better understand the biology to be able to identify those who are going to eventually develop recurrence and have the potential to die of their disease. Although there are well-established recurrence tests like the widely used Oncotype DX assay, incorporating features of CTCs, known to remain dormant and evade treatment, may improve upon their recurrence prediction, particularly in the longer term^8,20–22^. In this study, we aimed to determine if our CTC genomic signature has potential for predicting recurrence. Additionally, we aimed to assess whether the intratumour heterogeneity (ITH) of the CTC signature in primary breast tumors is related to recurrence risk.

We found strong differential expression in most of the selected CTC signature genes, with most being overexpressed in tumors, indicating a strong tumorigenic role of our targeted panel genes (Fig. 2A). A model with strong correlation to Oncotype DX, (expression CTC-sig model 10), could be trained from the data (Fig. 2B–D). Although the significance of the R value was affected by the sample size, group-level significance is not important in the context of administering the test clinically, which is done at the individual patient level. Notably, CTC-sig model 10 displayed robust performance by generating very similar median R values across a wide range of random subsets of various sizes. This is key for generating accurate and dependable recurrence scores for individual patients (Fig. 3A, B). As our signature was developed through isolating CTCs from patients with varying breast cancer subtypes, we believe our signature could be applied to other subtypes in which robust prognostic tests are lacking. This was observed through external validation in METABRIC survival data which demonstrated that CTC-sig model 10 could stratify long-term recurrence risk, (and not just Oncotype DX score), in a large independent cohort of all breast cancer subtypes. A significant difference in RFS as large as approximately 10% was achieved between low and high scoring patients over 20 years when using an optimal threshold for stratification (Fig. 3C). This was observed in all breast cancer subtypes as well as when filtering for only ER + /HER2− patients, indicating that the model has good transferability. We also observed that CTC signature genes were more strongly linked to RFS in later disease (>10 years after surgery) **(**Supplementary Fig. S4C**)**. This is consistent with the principle of minimal residual disease and tumor cell dormancy, whereby CTCs harbouring this signature disseminate and form micrometastases which can remain dormant for many years before manifesting into a clinically detectable late recurrence^25^. Together, these results underscore the potential of evaluating CTC expression in primary tumours. However, these results should be treated as approximations of model performance due to compatibility and scaling differences between our expression data and that of METABRIC.

The LP-WGS data revealed that CTC signature regions are mostly copy number neutral in primary tumors (Fig. 4A). This was expected because CTCs represent a rare subset of tumor cells whose genomic profiles are diluted during whole-tissue microdissection and DNA sequencing. In accordance with this, we have previously established that CTC signature gains are rare in breast carcinomas, only present in 3-4% of cases (*n* = 787) in TCGA^23^. Likely as copy number alterations may not substantially change RNA levels and gene expression captures actual functional activity in the tumor cells, which is often more directly linked to outcomes like proliferation, metastasis, or therapy resistance. Consequently, the generated copy number model only moderately correlated with Oncotype DX score, and gene expression is more appropriate to explore prognostication by CTCs in primary tumours. (Fig. 4B–D, Fig. 5A, B, Supplementary Figs. S7, S8). Despite only moderate performance against Oncotype DX, the copy number model performed relatively well in METABRIC RFS, in which it predicted RFS over 20 years almost as well as CTC-sig expression model 10 (Fig. 3C, Fig. 5C, Supplementary Fig. S9). Similarly, to the METABRIC gene expression, stratification results should be interpreted cautiously due to dataset differences.

We also evaluated CTC signature ITH in primary ER + /HER2− tumors with Multiplex-FISH. Results from 108 patients indicate that, higher ITH of select CTC signature regions is correlated with a higher risk of recurrence as determined by both Oncotype DX and by overall grade (Fig. 6). This is consistent with our previous finding that CTC signature ITH is higher in the primary tumors of patients with distant metastases than those without and those with higher heterogeneity have poor outcomes^24^. Notably, when stratifying patients by median ITH, the range of ITH scores in the lower half of the distribution was much smaller than in the upper half, which suggests that there may be a distinct subtype within ER + /HER2− breast cancer with greater intra-patient homogeneity (Supplementary Fig. S10). Indeed, molecular subtyping of ER + /HER2− breast cancer has led to further subclassification into luminal A and B subtypes with prognostic implications^26–28^. While these findings are exploratory, they highlight the potential of CTC signature ITH to capture intratumoral diversity and that could eventually inform risk stratification.

There are a few limitations to this study that should be noted and addressed before clinical utility of the signature can be explored. The same cohort was used for development and validation; we were limited to the number of patients that could be included that had a complete set of tumors and normal blocks and had enough material to undergo sequencing. Additionally, this is a single-centre study and the majority of cases at our institution are low Oncotype DX scoring which may have diluted our results. Future studies will need to include other institutions in order to fully understand the implications and correlation of this signature with a broader range of Oncotype DX scores. Second, Oncotype DX was used as a surrogate for outcome information due to challenges in obtaining comprehensive long-term survival data. The score should be validated on a large group of patients with 20+ years of follow-up data. Although the METABRIC dataset has extensive follow-up data, these cases do not reflect the modern practice of patient selection for adjuvant therapy based on Oncotype DX score for recurrence, and high-risk patients may not have received adjuvant therapy that is standard today. This difference may limit the applicability of our findings to current clinical management, and further validation in contemporary cohorts is warranted. Finally, the results of our correlation and stratification of CTCs within the METABRIC data should be considered investigational rather than definitive. Differences in sequencing platforms (i.e., Nanostring expression vs. Whole Transcriptome), and as such the approximation of model scores generated mean that the METABRIC analysis does not fully demonstrate the predictive ability of the signature. Future validation in independent cohorts using the same sequencing methods is warranted to establish reproducibility and clinical relevance.

In summary, this study outlines the importance of rare cell CTC signatures in recurrent ER + /HER2− breast cancer and emphasizes the role of these genes in tumour behaviour. The integration of our CTC signature into expression and copy number-based models demonstrates potential of our expression signature as a predictor of recurrence risk. The expression-based CTC-sig model 10, showing strong correlation to Oncotype DX score, proved to be particularly useful for stratifying long-term RFS in METABRIC data. Furthermore, our Multiplex-FISH analysis revealed a positive correlation between ITH of select CTC signature regions and recurrence risk, highlighting the importance of considering spatial tumor biology when assessing breast cancer behaviour. Future studies need to be done on external and independent cohorts with complete long term survival data to determine the clinical significance of this promising signature.

## Methods

### Patient Samples

This research was conducted in accordance with the Declaration of Helsinki. Ethical approval was obtained from the University Health Network Research Ethics Board, Toronto, Canada (UHN REB: 18-5132). Archival samples used in this study met the requirements for a waiver of consent, as determined by the institutional research ethics board. All samples were used in accordance with institutional ethics committee approval. The cohort comprised primary tumor formalin-fixed paraffin embedded (FFPE) tissue from 194 treatment-naïve female patients with ER + /HER2− receptor status who underwent recurrence testing with Oncotype DX between the years of 2007 to 2018 at the Princess Margaret Cancer Centre (Toronto, Canada) as standard of care. No preoperative (neoadjuvant) chemotherapy or endocrine therapy was administered to any of the patients. In Ontario (Canada), the decision to pursue Oncotype DX testing was made by the treating medical oncologist and patient based on patient age, primary tumor size, and grade. If needed, the test is reimbursed through Ontario’s publicly funded healthcare system. To investigate ITH a complete patient set included two tumor blocks from spatially separate areas of the primary tumor and a matched normal from adjacent tissue. Hematoxylin and eosin (H&E) staining was performed at the Pathology Research Program Laboratory (UHN), and carcinoma region annotation was done by qualified pathologists with additional training in breast pathology (Fig. 1).

### DNA/RNA Isolation from Tumor-normal samples

Ten 5 µm thick whole-tissue FFPE sections underwent deparaffinization in three 5 min xylene baths and rehydration in an ethanol series (100%, 95%, 70%, 5 minutes each). Slides were submerged in hematoxylin for 40 s for tissue visualization, followed by distilled water (dH_2_O) washes. Deparaffinized and stained sections were submerged in dH_2_O until microdissection. Sections were aligned with the corresponding pathologist-annotated H&Es under a light microscope, and tissue was collected with 18-gauge needles and stored in dH_2_O. Matched normal samples were macrodissected in their entirety for maximum yield. DNA and total RNA purification from macrodissected tissues was done with the AllPrep DNA/RNA FFPE Kit by QIAGEN, following the manufacturer’s instructions^29^. Samples were stored at -20 °C.

### CTC signature gene expression analysis

Genes in the 90 CTC signature regions were annotated in Ensembl BioMart. Gene Ontology identified top annotated genes associated with breast cancer pathways, cancer pathways, DNA repair, proliferation, and cell cycle. KEGG Pathway analysis ensured coverage of major breast cancer signaling pathways, yielding a final list of 89 CTC-related genes. Additionally, 24 breast cancer genes outside CTC regions but common to prognostic panels (PAM50, Lehmann’s classification, NanoString’s Breast360) were included. Fourteen EMT-related genes were added for their role in metastasis and CTC formation, and 5 housekeeping genes for normalization. The full list of genes can be found in Supplementary Table S4. A custom NanoString nCounter CodeSet was used to assess expression of these genes at the Princess Margaret Genomics Centre, Toronto, Canada (PMGC). RNA integrity and yield were determined with an Agilent 2100 Bioanalyzer, and 300 ng per sample was used for expression analysis due to the degraded state of FFPE RNA. Tumor samples that did not yield 300 ng of RNA were excluded. The NanoString nCounter assay was carried out as per manufacturer instructions and included the standard QC measures outlined in the nSolver user manual, excluding or repeating flagged samples^30,31^.

### CTC copy number analysis

CTC signature copy number was evaluated at the PMGC. DNA samples were assessed for quality and yield with an Invitrogen Qubit 4 Fluorometer. The NEBNext® FFPE DNA Repair v2 Module (E7360L) was used prior to library preparation to repair FFPE DNA. Low-pass whole genome sequencing (LP-WGS) was conducted on an Illumina NovaSeq 6000 System using paired-end 150 bp reads to achieve at least 1× coverage. Sequencing was conducted with 100 ng of DNA or 40 ng of DNA for low-yield samples. To ensure coverage was consistent in lower input samples, 3 samples were compared at 40 ng versus 100 ng (Supplementary Fig. S5). Samples with a yield lower than 40 ng of DNA were excluded. The Genome Reference Consortium Human Build 3 (GRCh38) was used for alignment using bwa-mem. Copy number was determined with ichorCNA by the Broad Institute; an R tool which implements the hidden Markov Model to segment the genome, assess tumor fraction, and predict copy number for each segment, from LP-WGS data^32,33^. A panel of normals was constructed from all sequenced matched-normal samples (*n* = 55) and used as a baseline reference of ploidy for copy number aberrations (CNA) calling in 1 megabase (Mb) segments of the genome. Samples with a low tumor fraction (<0.15) and/or high GC-Map MAD (>0.15) were excluded.

### Prognostic model generation

NanoString data was processed in R (v. 4.2.0) for differential gene expression analysis using NanoStringDiff (v. 1.28.0; Bioconductor). For prognostic model generation, gene expression was normalized to the geometric mean of 5 housekeeping (HK) genes: *PUM1, GUSB, GAPDH, POLR2A*, and *PSMC4* within each sample. Normalized tumor samples were randomly split into 60% training (*n* = 203) and 40% testing (*n* = 136) groups, repeated 10 times. Generalized linear models using Least Absolute Shrinkage and Selection Operator (LASSO) (glmnet v. 4.1-8) were created on each training subset against Oncotype DX scores and applied to the corresponding testing subset^34,35^. Ten-fold cross-validation was used to optimize the models, and the best model iteration was selected. Model robustness was validated on 1000 random subsets from 10% to 100% of tumor samples.

Whole genome copy number calls from ichorCNA were filtered for segments overlapping with CTC signature regions, totaling 123 segments. Stepwise feature selection (R stats package v. 4.2.0) generated a model against Oncotype DX scores^36^. Validation was done on 1000 random subsets as previously. LASSO regressions were trained on 60% training (*n* = 196) and 40% testing (*n* = 131) groups, repeated 1000 times with cross-validation. Two models with the highest correlation and significance to Oncotype DX scores were selected, and their robustness was validated on 1000 random subsets.

### External validation

All generated models were externally validated in the Molecular Taxonomy of Breast Cancer International Consortium (METABRIC) dataset in which recurrence and survival data is available up to 30 years after diagnosis^37^. Due to compatibility issues of the data, METABRIC gene expression was normalized to the same 5 HK genes as the expression model and the model was applied using the signs (+/−) only, without gene coefficients. For the copy number models, genes within model regions were annotated and used for score generation in the METABRIC data which is available at the gene level rather than for genomic regions. Genes for which copy number data was not available were excluded.

### Intratumour heterogeneity quantification

Multispectral fluorescence in situ hybridization (Multiplex-FISH) was used to quantify the copy number of 5 CTC genomic regions and a control centromeric region at single cell level and counts were used to calculate an ITH score (∆H) using the Shannon index, as described in detail in our previous work^24^. In brief, ∆H was calculated as the net difference in heterogeneity scores between spatially separate regions of the same tumor. Genomic regions evaluated for this study were *KLK10, MUC16, CCNE1, TGF-β1, BSG*, and chromosome 19 centromere. Using the same tumours sent for Oncotype testing tissue microarray (TMA) containing 0.6 mm diameter cores of all samples (2 tumor, 1 normal per patient) with 1 mm spacing was constructed. Cores were randomized by row and column for statistical validity. TMA construction was carried out using a tissue-arraying instrument (TMArrayer™).

### Statistics

The Pearson correlation coefficient was calculated for comparison between CTC signature expression, copy number score and Oncotype scores (α = 0.05). An optimal threshold determined using the ‘surv_cuptoint()’ function of the R package ‘survminer’ was used to stratify patients into low- and high-risk groups in the METABRIC expression and copy number cohorts^38^. For M-FISH normalization and to correct for tissue thickness, a 99% confidence interval for calling “true” single-gain clones and a global false positivity rate for tumor sample inclusion were calculated as detailed previously (Supplementary Tables S5-6)^24^. To determine the difference between Oncotype score, clinicopathological variables with our ITH score a Wilcoxon-rank sum test was used (α = 0.05).

## Supplementary information

Supplementary Information

## Data Availability

The datasets generated and/or analyzed during the current study are not publicly available due to the sensitive nature of the clinical data and restrictions imposed by the institutional research ethics board, de-identified data are available from the corresponding author (S.J. Done) on reasonable request.
