# Supplementary material for: The role of circulating tumor cell-associated genes in the progression of estrogen receptor-positive breast cancer
Source: NPJ Breast Cancer. 2025 Dec 15;12:11. doi: 10.1038/s41523-025-00874-0 (PMC12800032; doi:10.1038/s41523-025-00874-0)
Supplement: Supplementary file 1 — Supplementary Information [file 41523_2025_874_MOESM1_ESM.pdf]

Supplementary Figures

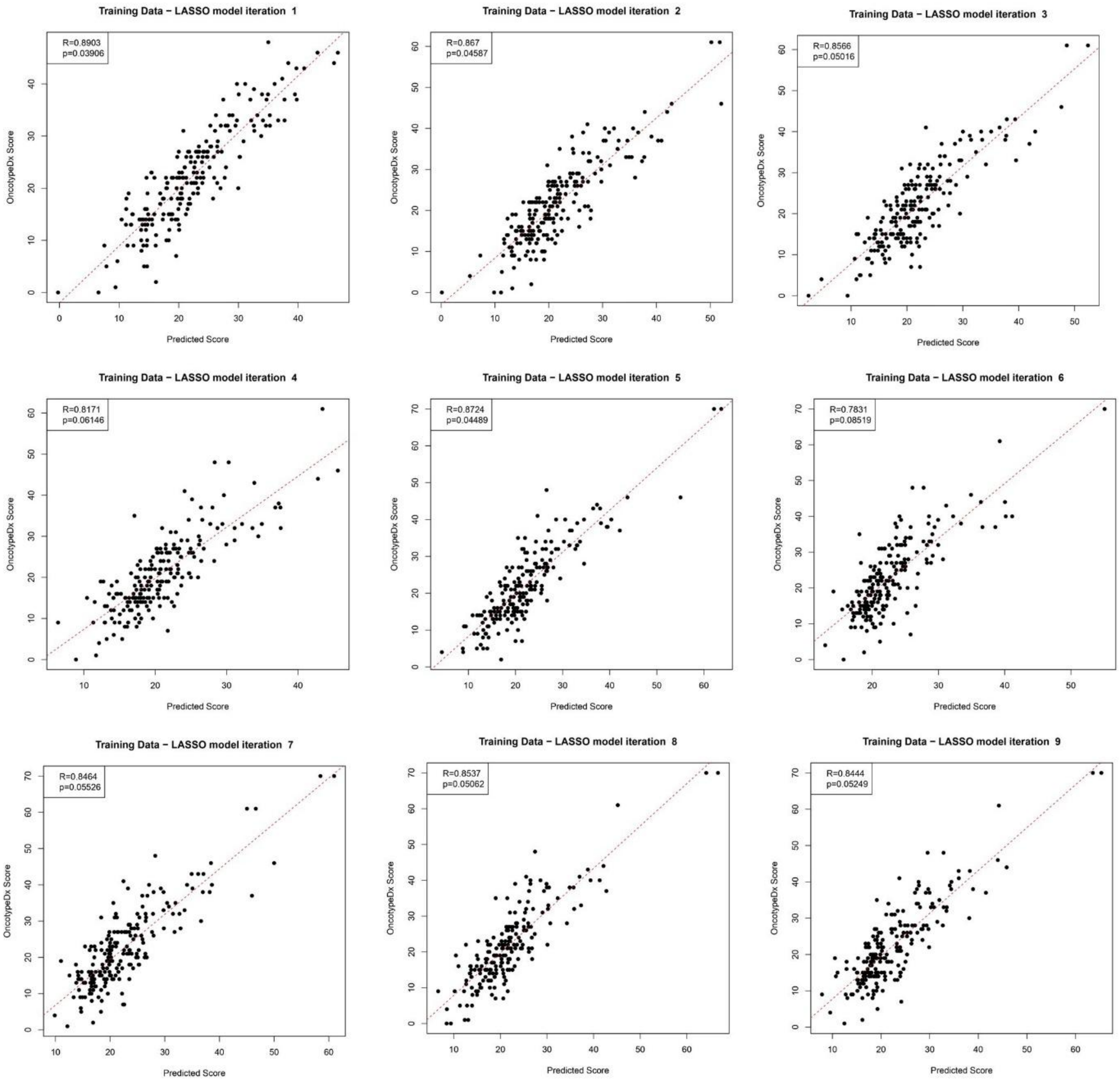

Supplementary Figure S1. LASSO expression-based model iterations in random subsets of 60% training samples (n = 203).

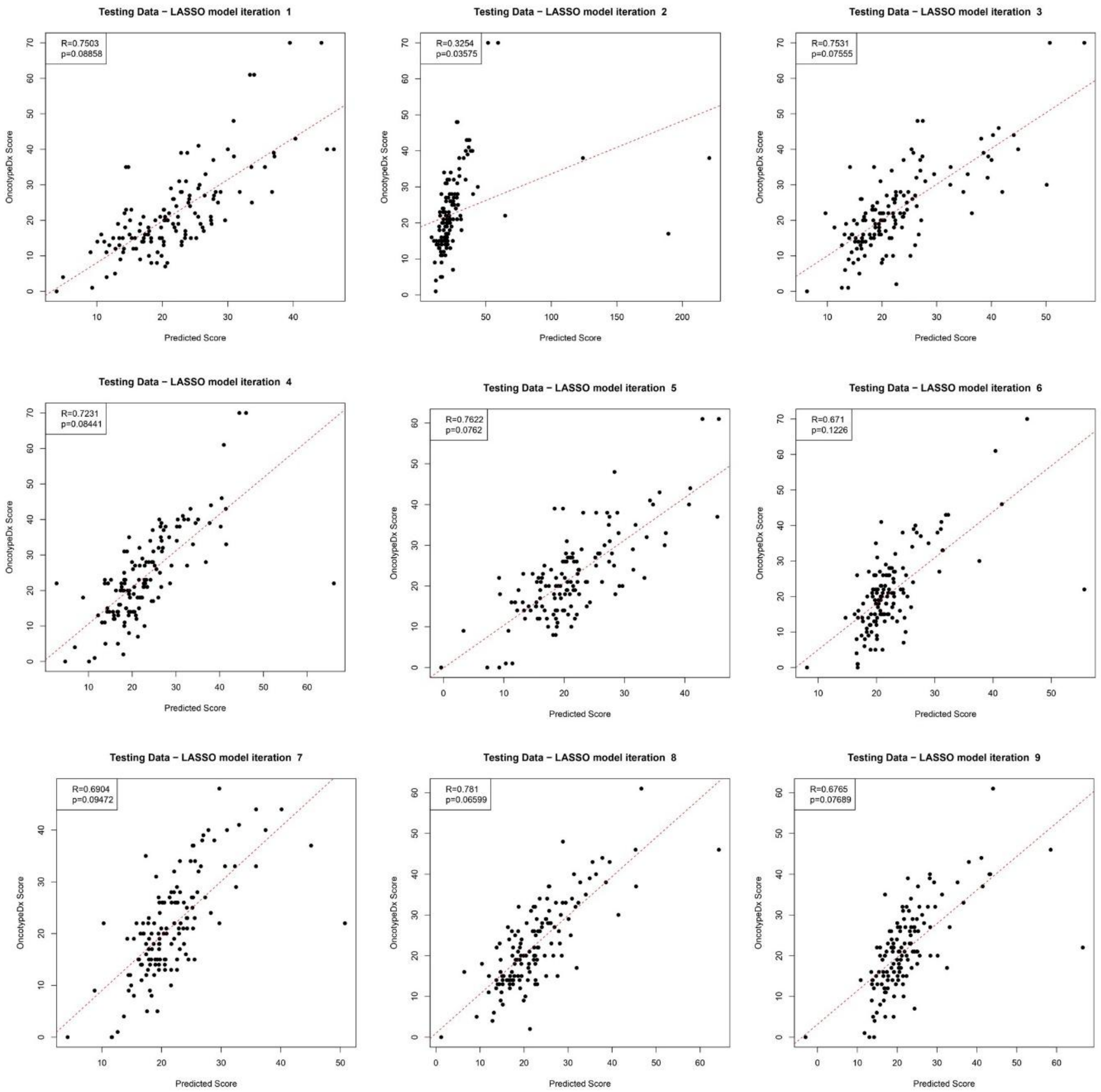

Supplementary Figure S2. LASSO expression-based model iterations in random subsets of 40% testing samples (n = 136), corresponding to training subsets.

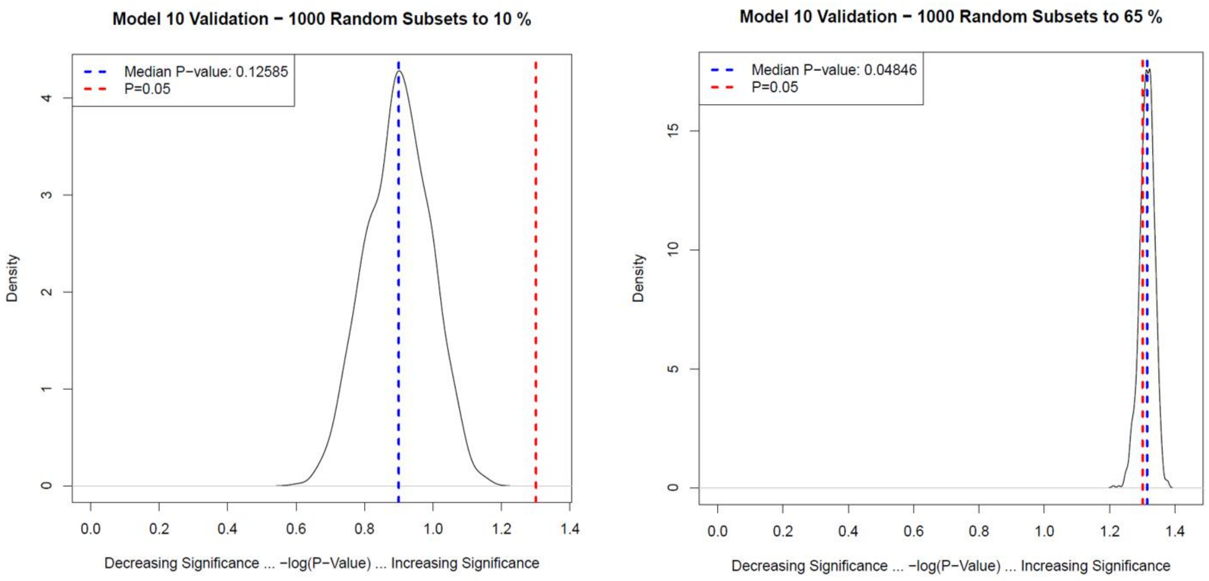

Supplementary Figure S3. Expression-based model 10 validation p-values. One thousand random subsets, ranging from 10% to 100% of all tumor samples, were generated to assess expression-based model 10 performance. Distributions of p values for 1000 subsets at 10% and 65% are displayed, with median p indicated by a blue dotted line and p = 0.05 indicated by a red dotted line.

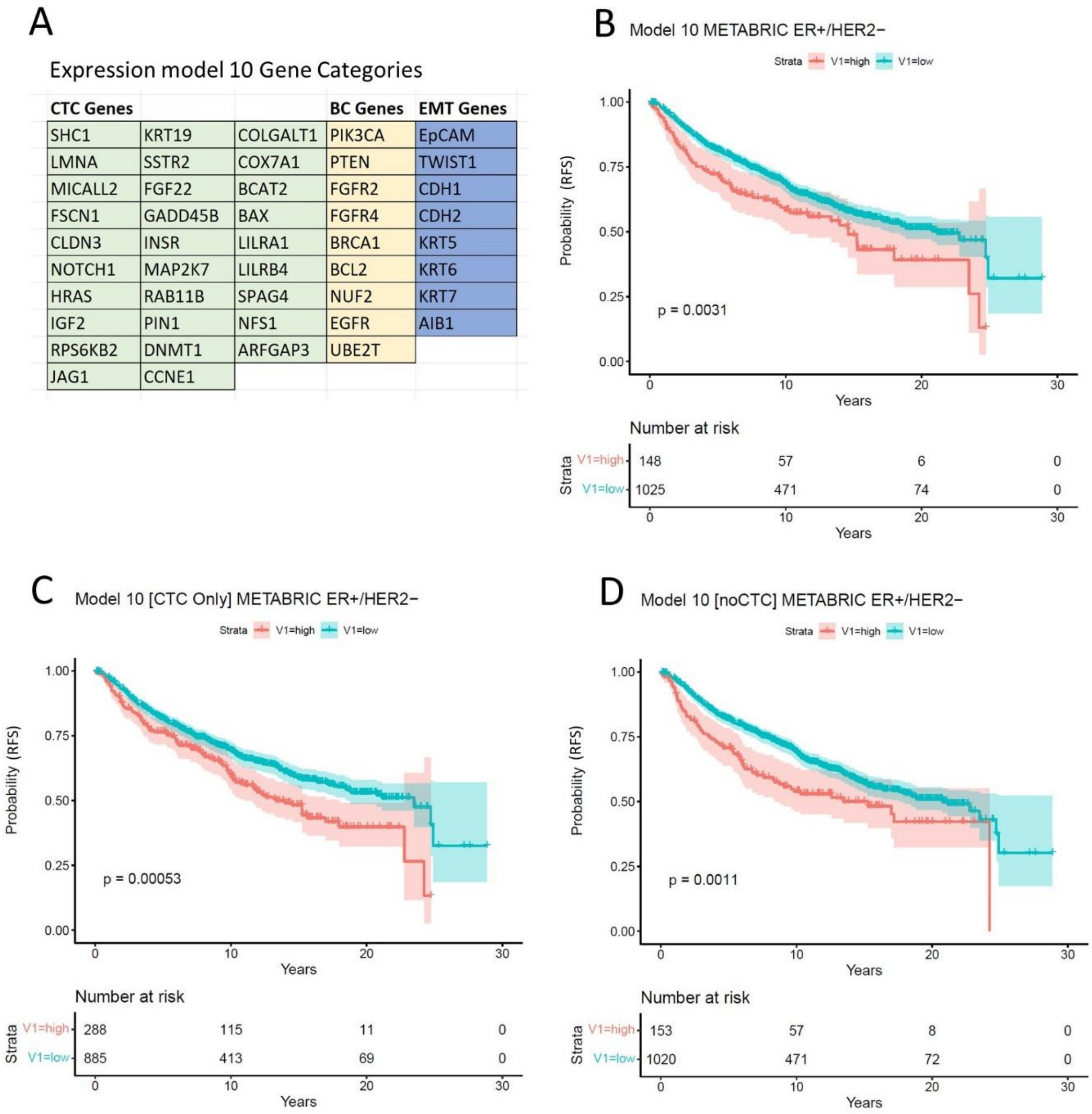

Expression model 10 Gene Categories

CTC Genes			BC Genes	EMT Genes
SHC1	KRT19	COLGALT1	PIK3CA	EpCAM
LMNA	SSTR2	COX7A1	PTEN	TWIST1
MICALL2	FGF22	BCAT2	FGFR2	CDH1
FSCN1	GADD45B	BAX	FGFR4	CDH2
CLDN3	INSR	LILRA1	BRCA1	KRT5
NOTCH1	MAP2K7	LILRB4	BCL2	KRT6
HRAS	RAB11B	SPAG4	NUF2	KRT7
IGF2	PIN1	NFS1	EGFR	AIB1
RPS6KB2	DNMT1	ARFGAP3	UBE2T	
JAG1	CCNE1			

Supplementary Figure S4. LASSO expression model 10 by gene category. **A.** Breakdown of 46 model 10 genes by CTC signature vs non-CTC signature (BC genes + EMT genes). **B-D.** Kaplan Meier survival curves of model 10, model 10 CTC signature genes, and model 10 non-CTC signature genes in ER+/HER2- METABRIC patients. Models were applied without coefficients and used to stratify patients into low (turquoise) and high (orange) risk groups. Shaded areas represent a 95% confidence interval and the log-rank p-value is <0.01.

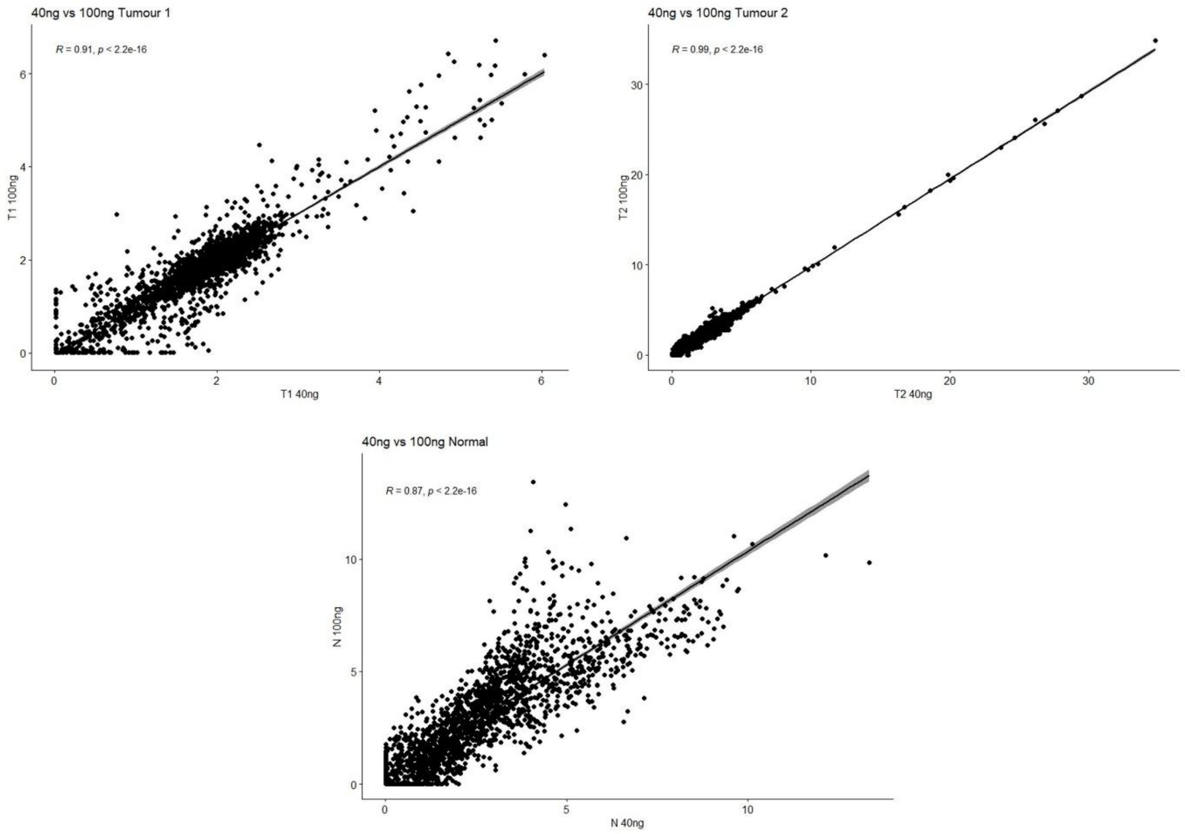

Supplementary Figure S5. Examining ichorCNA copy number calls in 40 ng versus 100 ng sequencing runs. A comparative analysis of copy number estimates for 1 Mb segments across the whole genome in three samples (2 tumors and 1 normal) was made with Pearson Correlation. The sequencing was conducted at both 40 ng and 100 ng of DNA, and the results processed with ichorCNA. Pearson $R \geq 0.87$ was achieved in all comparisons ($p < 2.2^{-16}$).

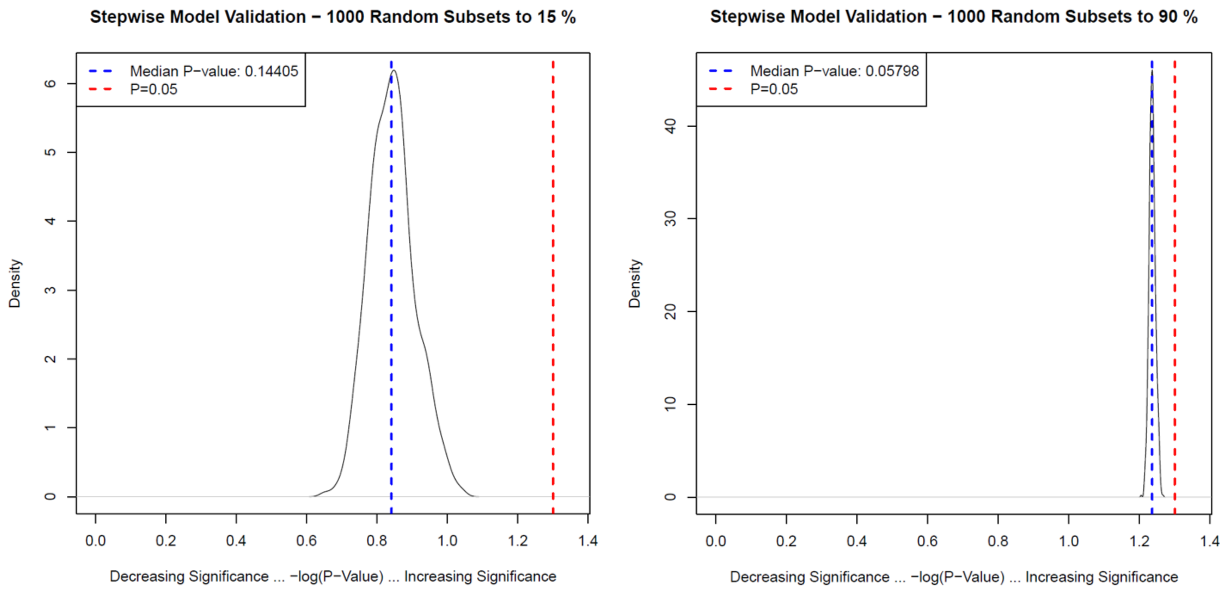

Supplementary Figure S6. Copy number-based model validation p-values. One thousand random subsets, ranging from 15% to 100% of all tumor samples, were generated and used to test the performance of the stepwise copy number model. Distributions of p values for 1000 subsets at 15% and 90% are displayed, with median p indicated by a blue dotted line and p = 0.05 indicated by a red dotted line.

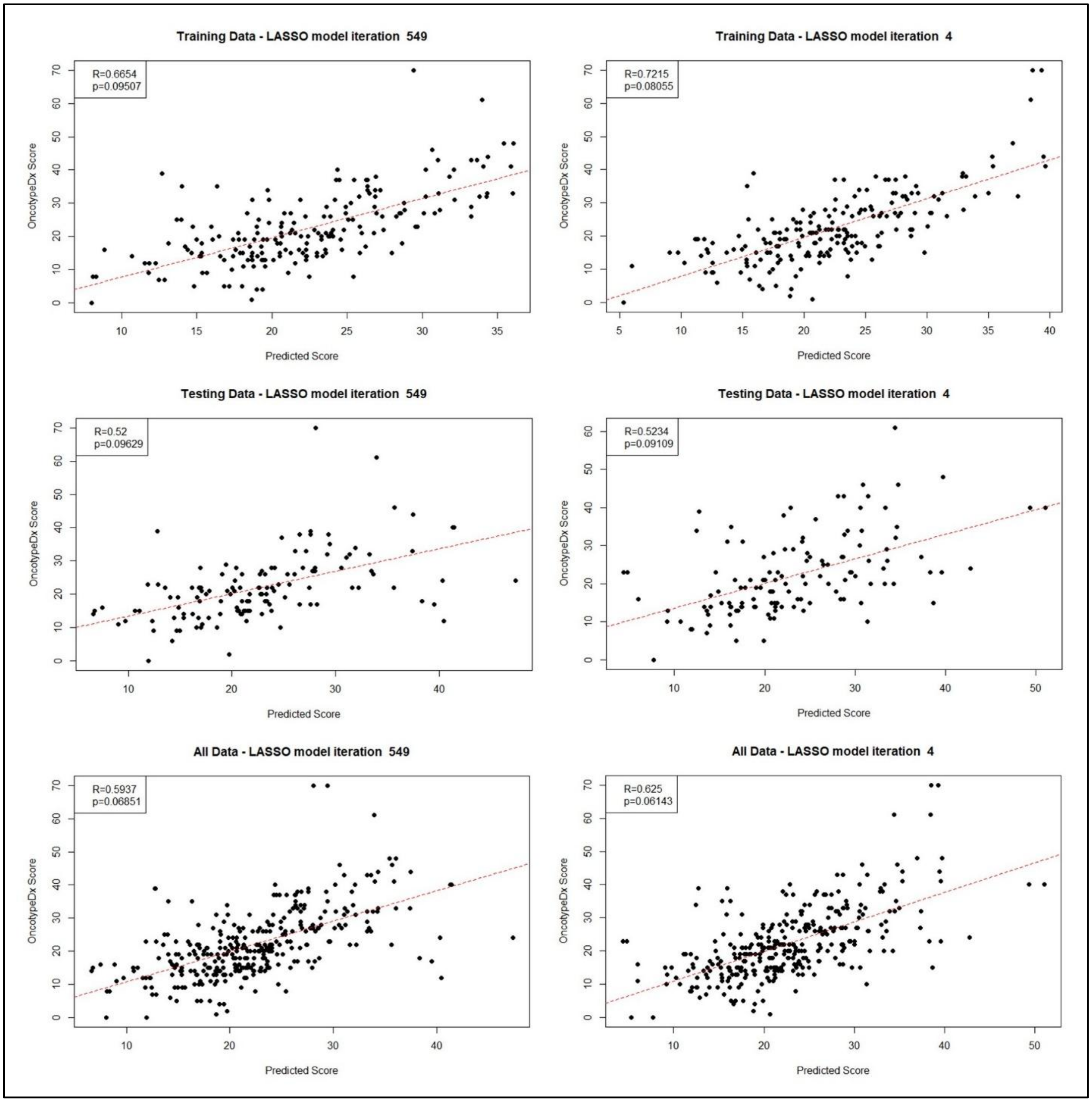

Supplementary Figure S7. Evaluation of LASSO copy number models 4 and 549 against Oncotype DX. Models 4 and 549 were selected for having the best performance of 1000 LASSO model iterations. The Pearson R correlation coefficient and p value of each model in subsets of 60% training data (n = 196), 40% testing data (n = 131), and all data (n = 327) are displayed.

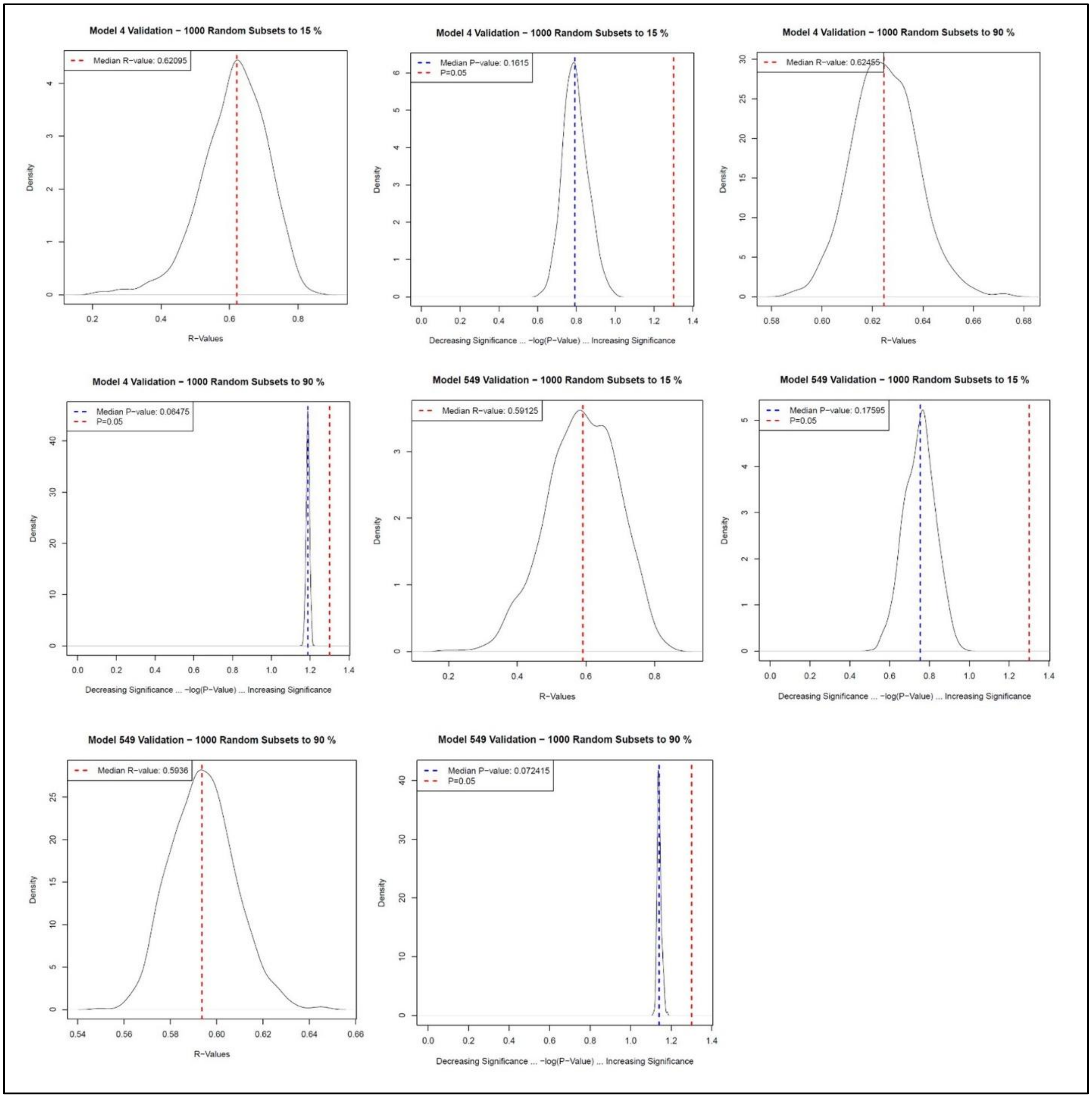

Supplementary Figure S8. Validation of LASSO copy number models 4 and 549 in 1000 random subsets. Subsets varied in size from 15% to 100% of all tumor samples. The distributions of correlation coefficients (R) and p-values achieved by the models in 1000 subsets to 15% and 90% are shown. Dotted lines in each plot indicate the median R and p-values, and the p = 0.05 threshold (refer to legends on the figure).

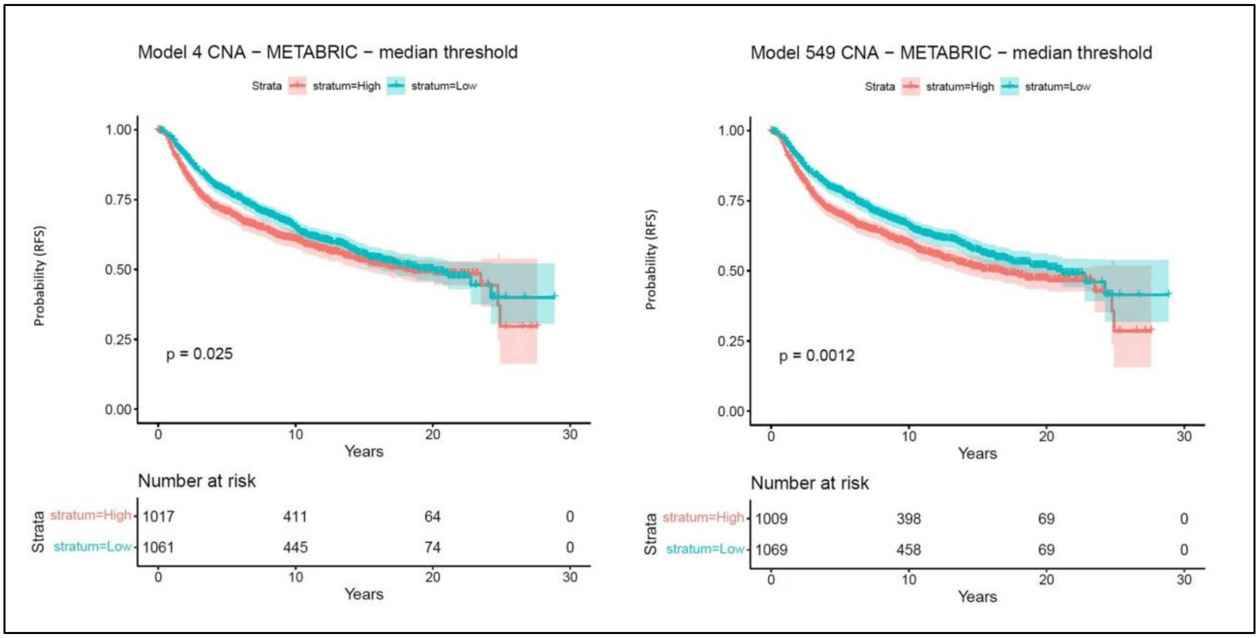

Supplementary Figure S9. Kaplan-Meier survival analysis depicting the performance of LASSO copy number models 5 and 549 in METABRIC data. The models were applied to METABRIC copy number data, utilizing annotated genes from the CTC regions of each model. The resulting scores were plotted against recurrence-free survival (RFS) in years. Patients were categorized into low (turquoise) and high (orange) score groups based on the median score of each model. Shaded areas represent a 95% confidence interval, and the number of patients in each group is displayed over time at the bottom. Log-rank p-values of 0.025 and 0.0012 were obtained for models 4 and 549, respectively.

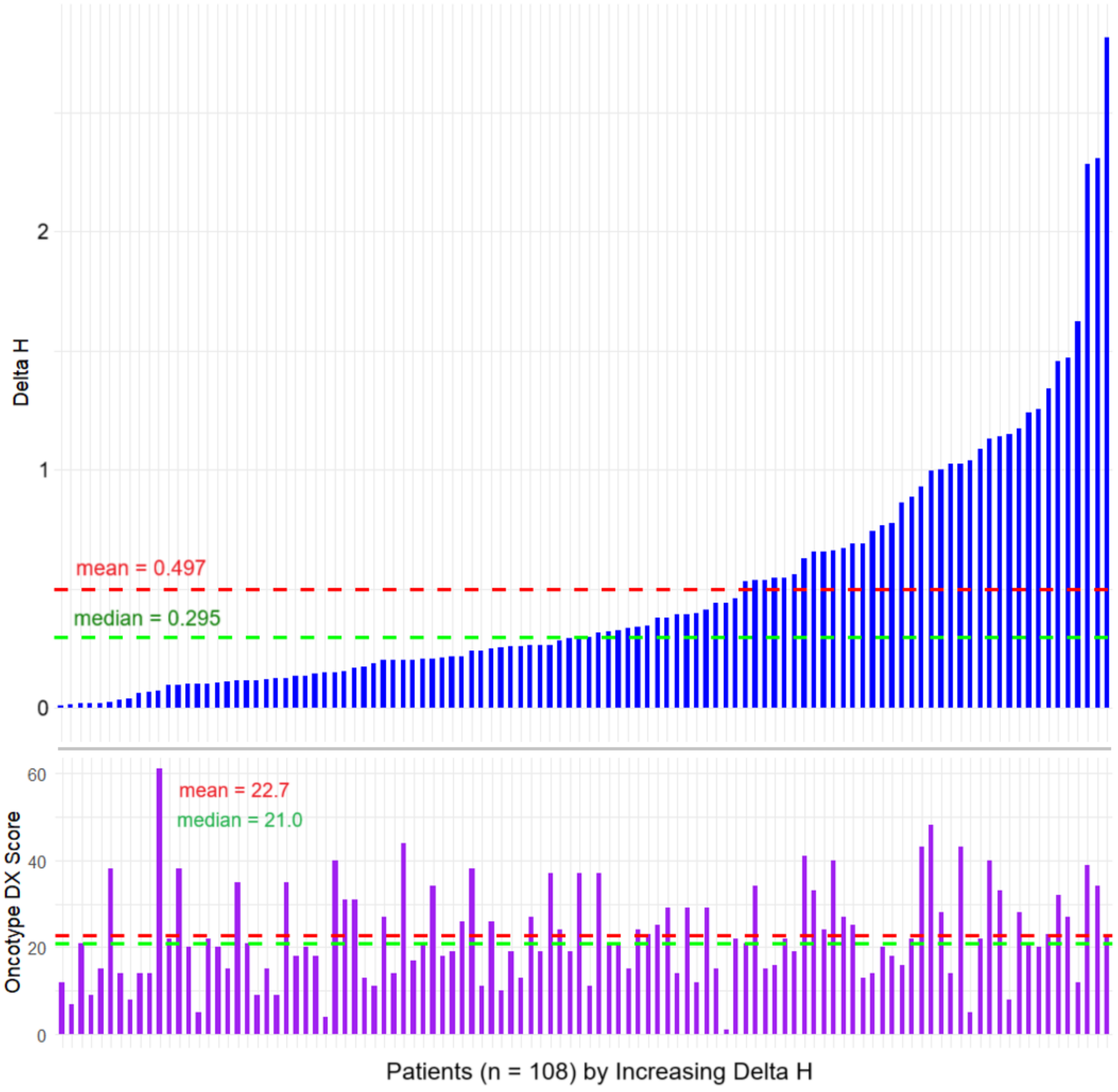

Supplementary Figure S10. Distribution analysis of ΔH and Oncotype DX scores in 108 patients. Patients are ordered based on ascending ΔH scores. The red dotted line marks the mean ΔH score at 0.50, and the green dotted line indicates the median at 0.30. The mean and median Oncotype DX scores are 23 (red dotted line) and 21 (green dotted line).

Supplementary Tables

Supplementary Table S1. Expression-based model 10. LASSO regression was used to train models against Oncotype DX recurrence scores and the 10th iteration of the model, consisting of 46 genes, was selected for having the best performance. Genes are listed by descending absolute weight factor.

+31.9[CCNE1] -24.1[LILRA1] -23.3[FGF22] -14.3[SSTR2] +13.0[CDH2] +10.9[PIK3CA] -9.58[BCL2] +9.55[NUF2] +9.45[DNMT1] +8.99[SPAG4] -8.62[MICALL2] -6.71[BCAT2] +6.35[NOTCH1] -5.87[MAP2K7] -5.50[COX7A1] +5.03[FSCN1] -4.97[PTEN] +4.70[LILRB4] -4.39[RAB11B] +3.70[UBE2T] +3.53[BAX] +3.25[KRT6] +3.14[FGFR4] -2.88[HRAS] -2.25[ARFGAP3] 2.05[AIB1] +1.95[KRT5] +1.81[NFS1] +1.74[JAG1] +1.65[CDH1] -1.52[INSR] +1.33[EpCAM] +1.31[CLDN3] -1.10[RPS6KB2] +0.971[FGFR2] +0.951[TWIST1] +0.593[EGFR] +0.591[KRT7] -0.427[KRT19] -0.312[BRCA1] +0.268[COLGALT1] +0.171[SHC1] +0.168[PIN1] +0.0951[GADD45B] +0.0103[LMNA] -0.00332[IGF2] +21.8

Supplementary Table S2. Copy number models generated with stepwise feature selection or LASSO from CTC signature copy number data.

Stepwise Feature Selection Model	+48.87[CTCreg.11] -44.3[CTCreg.77] +42.82[CTCreg.80] -35.47[CTCreg.12] -30.33[CTCreg.79] -30.09[CTCreg.39] +30.06[CTCreg.67] +29.74[CTCreg.118] -28.77[CTCreg.92] -28.49[CTCreg.83] +27.59[CTCreg.89] -27.29[CTCreg.10] +23.57[CTCreg.86] +22.62[CTCreg.40] +17.82[CTCreg.113] -17.7[CTCreg.111] +17.57[CTCreg.73] -17.48[CTCreg.119] -16.29[CTCreg.47] -12.5[CTCreg.20] +12.03[CTCreg.28] +10.95[CTCreg.100] -6.36[CTCreg.26] -5[CTCreg.9] +4.76[CTCreg.4] +26.29
LASSO Model 4	+34.14[CTCreg.11] -23.65[CTCreg.10] -22.46[CTCreg.90] +21.07[CTCreg.80] -19.36[CTCreg.47] -18.4[CTCreg.39] +17.68[CTCreg.40] +17[CTCreg.57] -16.21[CTCreg.59] +15.78[CTCreg.67] -12.38[CTCreg.119] -10.74[CTCreg.103] -9.98[CTCreg.20] -9.36[CTCreg.12] +9.09[CTCreg.100] -7.56[CTCreg.31] +7.41[CTCreg.122] -7.4[CTCreg.18] +7.33[CTCreg.113] -7.07[CTCreg.77] +6.29[CTCreg.28] +5.76[CTCreg.27] +5.13[CTCreg.115] -4.2[CTCreg.26] +3.92[CTCreg.4] +3.16[CTCreg.118] -2.97[CTCreg.87] +2.79[CTCreg.33] +2.78[CTCreg.98] +2.21[CTCreg.99] +1.79[CTCreg.117] -1.78[CTCreg.108] +1.58[CTCreg.6] +1.46[CTCreg.55] +1.44[CTCreg.86] -1.41[CTCreg.2] -1.24[CTCreg.93] +0.94[CTCreg.69] -0.83[CTCreg.106] -0.79[CTCreg.37] +0.68[CTCreg.54] +0.68[CTCreg.73] +0.62[CTCreg.8] -0.55[CTCreg.105] +0.43[CTCreg.58] +0.33[CTCreg.21] +0.31[CTCreg.22] +0.23[CTCreg.74] -0.18[CTCreg.91] +0.02[CTCreg.56] +0.02[CTCreg.76] +0.01[CTCreg.52] +21.7
LASSO Model 549	+27[CTCreg.11] +18.1[CTCreg.52] +17.07[CTCreg.80] -16.85[CTCreg.10] -15.68[CTCreg.65] +13.85[CTCreg.70] -12.53[CTCreg.47] -9.96[CTCreg.37] +9.13[CTCreg.28] +8.93[CTCreg.33] -7.47[CTCreg.31] -7.05[CTCreg.16] +5.74[CTCreg.8] +5.72[CTCreg.56] -5.63[CTCreg.90] -4.92[CTCreg.79] -4.75[CTCreg.39] -4.37[CTCreg.61] +4.06[CTCreg.2] -3.77[CTCreg.19] +3.77[CTCreg.27] +3.65[CTCreg.115] +3.5[CTCreg.113] +3.34[CTCreg.78] -3.1[CTCreg.66] -2.8[CTCreg.7] -2.64[CTCreg.20] -2[CTCreg.21] -1.91[CTCreg.29] -

	1.76[CTCreg.103] +1.02[CTCreg.67] -0.72[CTCreg.22] -0.68[CTCreg.12] -0.53[CTCreg.104] +0.5[CTCreg.4] -0.22[CTCreg.121] +0.2[CTCreg.77] +0.16[CTCreg.53] +0.16[CTCreg.98] -0.11[CTCreg.91] -0.01[CTCreg.26] +0.01[CTCreg.54] +22.73

Supplementary Table S3. CTC region to genomic position conversion table for copy number model regions.

Region Name	Chr	Start (bp)	End (bp)	Region Name	Chr	Start (bp)	End (bp)
CTCreg.1	1	1828508	3571573	CTCreg.63	19	11647783	11669900
CTCreg.2	1	1828508	3571573	CTCreg.64	19	11748055	11751088
CTCreg.3	1	154963903	155224815	CTCreg.65	19	12688300	13354750
CTCreg.4	1	154963903	155224815	CTCreg.66	19	12059529	12255322
CTCreg.5	1	155247948	156217829	CTCreg.67	19	12688300	13354750
CTCreg.6	1	155247948	156217829	CTCreg.68	19	13897508	14127977
CTCreg.7	7	1172124	1526248	CTCreg.69	19	13893212	13897508
CTCreg.8	7	99728602	100805098	CTCreg.70	19	14127977	14621779
CTCreg.9	8	144748397	145258668	CTCreg.71	19	13897508	14127977
CTCreg.10	9	130341832	132263422	CTCreg.72	19	16533859	17171711
CTCreg.11	9	130341832	132263422	CTCreg.73	19	17604798	19933536
CTCreg.12	9	130341832	132263422	CTCreg.74	19	16533859	17171711
CTCreg.13	9	133754159	134008327	CTCreg.75	19	17247596	17604798
CTCreg.14	9	133754159	134008327	CTCreg.76	19	17171711	17247596
CTCreg.15	9	134008327	134427749	CTCreg.77	19	17604798	19933536
CTCreg.16	9	135952412	137342684	CTCreg.78	19	17604798	19933536
CTCreg.17	9	135952412	137342684	CTCreg.79	19	35962468	36806907
CTCreg.18	9	135952412	137342684	CTCreg.80	19	35962468	36806907
CTCreg.19	11	198510	2330394	CTCreg.81	19	36874368	37003268
CTCreg.20	11	198510	2330394	CTCreg.82	19	36806907	36874368
CTCreg.21	11	66088827	67455721	CTCreg.83	19	36874368	37003268
CTCreg.22	12	121601130	122376372	CTCreg.84	19	38822004	39533740
CTCreg.23	12	121601130	122376372	CTCreg.85	19	38676607	38822004
CTCreg.24	16	61588	2269598	CTCreg.86	19	38822004	39533740
CTCreg.25	16	61588	2269598	CTCreg.87	19	40932895	41305936
CTCreg.26	16	2269598	3147134	CTCreg.88	19	40932895	41305936
CTCreg.27	16	69119444	70586226	CTCreg.89	19	45114963	46335814
CTCreg.28	16	85028133	85431985	CTCreg.90	19	46954437	48769480
CTCreg.29	17	41247881	41270183	CTCreg.91	19	45114963	46335814
CTCreg.30	17	41270183	41705015	CTCreg.92	19	46954437	48769480
CTCreg.31	17	72782884	74534518	CTCreg.93	19	46954437	48769480
CTCreg.32	17	72782884	74534518	CTCreg.94	19	48873884	49499933
CTCreg.33	17	72782884	74534518	CTCreg.95	19	48769480	48822048
CTCreg.34	19	329975	1540502	CTCreg.96	19	48822048	48866690
CTCreg.35	19	1541476	2145767	CTCreg.97	19	48866690	48873884
CTCreg.36	19	1540502	1541476	CTCreg.98	19	49499933	50531298
CTCreg.37	19	2145767	4742395	CTCreg.99	19	48873884	49499933
CTCreg.38	19	1541476	2145767	CTCreg.100	19	49499933	50531298
CTCreg.39	19	2145767	4742395	CTCreg.101	19	55622672	56124070
CTCreg.40	19	2145767	4742395	CTCreg.102	19	55536834	55622672
CTCreg.41	19	4975556	5917988	CTCreg.103	19	55622672	56124070
CTCreg.42	19	4742395	4822114	CTCreg.104	20	34974415	35518080
CTCreg.43	19	4901877	4975556	CTCreg.105	20	34974415	35518080
CTCreg.44	19	4841075	4893005	CTCreg.106	20	35518080	35526175
CTCreg.45	19	4822114	4841075	CTCreg.107	20	35526175	35528381
CTCreg.46	19	4893005	4901877	CTCreg.108	20	35528381	35649226
CTCreg.47	19	4975556	5917988	CTCreg.109	20	35649226	35702851
CTCreg.48	19	5917988	5926007	CTCreg.110	20	35702851	35717517
CTCreg.49	19	7903395	8218214	CTCreg.111	20	60667621	60965220
CTCreg.50	19	7480881	7771995	CTCreg.112	20	60965220	61206692
CTCreg.51	19	7266839	7480881	CTCreg.113	20	60965220	61206692
CTCreg.52	19	7903395	8218214	CTCreg.114	20	61206692	61515573
CTCreg.53	19	8389265	8659813	CTCreg.115	22	37933242	39905317
CTCreg.54	19	8222315	8389265	CTCreg.116	22	37933242	39905317
CTCreg.55	19	8659813	8778208	CTCreg.117	22	37933242	39905317
CTCreg.56	19	8218214	8222315	CTCreg.118	22	42669807	43327972
CTCreg.57	19	9839918	10194514	CTCreg.119	22	42669807	43327972
CTCreg.58	19	9689188	9839918	CTCreg.120	22	44925914	45215304
CTCreg.59	19	10194514	11647783	CTCreg.121	22	44925914	45215304
CTCreg.60	19	9839918	10194514	CTCreg.122	22	46659270	47032308
CTCreg.61	19	10194514	11647783	CTCreg.123	22	46659270	47032308
CTCreg.62	19	11669900	11748055				

Suplememntary Table S4. List of all genes in expression analysis. Genes are broken down into CTC signature genes (green), breast cancer genes (yellow), EMT genes (blue) and reference genes (orange).

Classification	Gene	Description	Classification	Gene	Description
CTC signature	HES5	hes family bHLH transcription factor 5	CTC signature	LILRA1	leukocyte immunoglobulin like receptor A1
	EFNA1	ephrin A1		LILRB4	leukocyte immunoglobulin like receptor B4
	SHC1	SHC adaptor protein 1		SBK2	SH3 domain binding kinase family member 2
	LMNA	lamin A/C		EPN1	epsin 1
	MICALL2	MICAL like 2		GDF5	growth differentiation factor 5
	FSCN1	Fascin Actin-Bundling Protein 1		SPAG4	sperm associated antigen 4
	FZD9	frizzled class receptor 9		NFS1	NFS1 cysteine desulfurase
	CLDN3	claudin 3		RBM39	RNA binding motif protein 39
	CLDN4	claudin 4		CDH4	cadherin 4
	MUC1	mucin 1, cell surface associated		PDGFB	platelet derived growth factor subunit B
	MIR25	MicroRNA 25		ARFGAP3	ADP ribosylation factor GTPase activating protein 3
	ABL1	ABL proto-oncogene 1, non-receptor tyrosine kinase		GRAMD4	GRAM domain containing 4
	VAV2	vav guanine nucleotide exchange factor 2		MIR1	microRNA 1
	RXRA	retinoid X receptor alpha		ARHGAP8	Rho GTPase activating protein 8
	NOTCH1	notch receptor 1		CCL24	C-C motif chemokine ligand 24
	HRAS	HRas proto-oncogene, GTPase		CCL25	C-C motif chemokine ligand 25
	IGF2	insulin like growth factor 2		CCL26	C-C motif chemokine ligand 26
	RPS6KB2	ribosomal protein S6 kinase B2		MUC16	mucin 16, cell surface associated
	ORAI1	ORAI calcium release-activated calcium modulator 1		KLK10	kallikrein related peptidase 10
	AXIN1	Protein Phosphatase 1, Regulatory Subunit 49		EPO	Erythropoietin
	PDPK1	3-phosphoinositide dependent protein kinase 1		GNB2	G Protein Subunit Beta 2
	MAPK3	mitogen-activated protein kinase 3		JAG1	Jagged Canonical Notch Ligand 1
	NQO1	NAD(P)H quinone dehydrogenase 1		CCNE1	cyclin E1
	ERBB2	erb-b2 receptor tyrosine kinase 2	Other common BC genes	PIK3CA	phosphatidylinositol-4,5-bisphosphate 3-kinase catalytic subunit alpha
	BSG	basigin (Ok blood group)		PTEN	phosphatase and tensin homolog
	CDC6	cell division cycle 6		FGFR2	fibroblast growth factor receptor 2
	NUMBL	NUMB like endocytic adaptor protein		FGFR4	fibroblast growth factor receptor 4
	SOCS7	suppressor of cytokine signaling 7		MKI67	Marker Of Proliferation Ki-67
	MIR181	MicroRNA 181		BIRC5	baculoviral IAP repeat containing 5
	ARHGAP23	Rho GTPase activating protein 23		CCNB1	Cyclin B1
	KRT20	Keratin 20		CCND1	Cyclin D1
	KRT19	keratin 19		MYBL2	MYB proto-oncogene like 2
	SSTR2	somatostatin receptor 2		BRCA1	BRCA1 DNA repair associated
	APC2	APC regulator of WNT signaling pathway 2		BRCA2	BRCA2 DNA repair associated
	FGF22	fibroblast growth factor 22		MMP11	matrix metallopeptidase 11
	SHC2	SHC adaptor protein 2		GRB7	growth factor receptor bound protein 7
	TCF3	transcription factor 3		ESR1	estrogen receptor 1
	GADD45B	growth arrest and DNA damage inducible beta		PGR	progesterone receptor
	MAP2K2	mitogen-activated protein kinase kinase 2		BCL2	B-cell lymphoma 2
	TICAM1	toll like receptor adaptor molecule 1		BAG1	BAG cochaperone 1
	NDUFA11	NADH:ubiquinone oxidoreductase subunit A11		MYC	MYC proto-oncogene, bHLH transcription factor
	INSR	insulin receptor		CACNG4	calcium voltage-gated channel auxiliary subunit gamma 4
	MCOLN1	mucolipin 1		CD44	CD44 molecule (Indian blood group)
	MAP2K7	mitogen-activated protein kinase kinase 7		NUF2	NUF2 component of NDC80 kinetochore complex
	CERS4	ceramide synthase 4		EGFR	epidermal growth factor receptor
	RAB11B	RAB11B, member RAS oncogene family		TP53	tumor protein p53
	PIN1	peptidylprolyl cis/trans isomerase, NIMA-interacting 1		UBE2T	ubiquitin conjugating enzyme E2 T
	DNMT1	DNA methyltransferase 1	EMT genes	EpCAM	epithelial cell adhesion molecule
	EPOR	erythropoietin receptor		TWIST1	twist family bHLH transcription factor 1
	RNASEH2A	ribonuclease H2 subunit A		TERT	telomerase reverse transcriptase
	PRKACA	protein kinase cAMP-activated catalytic subunit alpha		CDH1	cadherin 1
	F2RL3	F2R like thrombin or trypsin receptor 3		CDH2	cadherin 2
	USE1	unconventional SNARE in the ER 1		ZEB1	zinc finger E-box binding homeobox 1
	COLGALT1	collagen beta(1-O)galactosyltransferase 1		SNAI1	snail family transcriptional repressor 1
	PIK3R2	phosphoinositide-3-kinase regulatory subunit 2		SNAI2	snail family transcriptional repressor 2
	COX7A1	cytochrome c oxidase subunit 7A1		KRT5	Keratin 5
	ACTN4	actinin alpha 4		KRT6	Keratin 6
	DLL3	delta like canonical Notch ligand 3		KRT7	Keratin 7
	TGFB1	transforming growth factor beta 1		KRT14	Keratin 14
	MIR330	microRNA 330		KRT18	Keratin 18
	VASP	vasodilator stimulated phosphoprotein		NCOA3	nuclear receptor coactivator 3 (AIB1)
	FGF21	fibroblast growth factor 21	Reference/ HK genes	PUM1	Pumilio homolog 1
	BCAT2	branched chain amino acid transaminase 2		GUSB	Beta-glucuronidase
	PPP1R15A	Protein phosphatase 1 regulatory subunit 15A		GAPDH	Glyceraldehyde 3-phosphate dehydrogenase
	BAX	BCL2 associated X, apoptosis regulator		POLR2A	DNA-directed RNA polymerase II subunit RPB1
	MIR150	microRNA 150		PSMC4	Proteasome 26S subunit, ATPase 4

Supplementary Table S5. Establishing thresholds for true single gain clones. A 99% confidence interval was defined by setting a threshold at 3 standard deviations above the average count for each probe in normal cells, ensuring accurate identification of genuine single gain clones.

Gene region	Average count	Standard deviation	True gain threshold (99% CI)
19cen	1.425163235	0.895044641	4.110297157
KLK10	1.61702662	0.865621497	4.213891112
TGF-B1	1.44249121	0.866399562	4.041689896
MUC16	1.64816675	0.843742815	4.179395197
CCNE1	1.561165536	0.854246883	4.123906185
BSG	1.291405184	0.870234883	3.902109832

Supplementary Table S6. Determining false positivity rates for tumor core inclusion. The global false positivity rate, computed using normal samples, was derived by dividing the number of cells displaying any two gains (copy number ≥ 3n) by the total number of cells assessed. The resulting global false positivity rate was determined to be 10.6%. Total number of all 2 gain combinations is displayed.

	19CEN	KLK10	TGFB1	MUC16	CCNE1	BSG
19CEN		133	107	127	147	22
KLK10	133		132	184	167	30
TGFB1	107	132		128	113	27
MUC16	127	184	128		153	28
CCNE1	147	167	113	153		20
BSG	22	30	27	28	20	
